# Associations of the unhealthy plant-based diet index with hsCRP and BMI in Korean adults

**DOI:** 10.3389/fnut.2026.1836622

**Published:** 2026-08-04

**Authors:** Ara Yoo, Injoong Shin, Hyoyeon Son, Won Jang, Hyesook Kim

**Affiliations:** 1 Department of Food and Nutrition, Wonkwang University, Iksan, Jeollabuk-do, Republic of Korea; 2 Department of Food and Nutrition, Dongseo University, Busan, Republic of Korea; 3 Institute for Better Living, Wonkwang University, Iksan, Jeollabuk-do, Republic of Korea

**Keywords:** BMI, hsCRP, KNHANES, Korean adults, obesity, plant-based dietary index (PDI), unhealthy PDI (uPDI)

## Abstract

**Background:**

This study investigated the associations of the unhealthy Plant-based Diet Index (uPDI) with high-sensitivity C-reactive protein (hsCRP) and body mass index (BMI) using data from the 2022–2023 Korea National Health and Nutrition Examination Survey.

**Methods:**

The study included adults aged 19–64 years, excluding individuals with implausible energy intake (<500 or >5,000 kcal/day), underweight status, or missing data, resulting in a final sample of 5,610 participants. The uPDI was calculated by summing energy-adjusted, quintile-based scores across 19 predefined food groups. Elevated hsCRP was defined as ≥3 mg/L, and obesity as BMI ≥ 25 kg/m^2^. Survey-weighted regression analyses were performed after adjustment for demographic and lifestyle covariates.

**Results:**

Among women, higher uPDI scores were positively associated with both hsCRP (*p* < 0.001) and BMI (*p* < 0.001) in multivariable-adjusted linear regression models. Logistic regression analyses revealed that higher uPDI scores correlated with elevated hsCRP (odds ratio [OR]: 2.043; 95% confidence interval [CI], 1.298–3.217; P-trend = 0.006) and obesity (OR: 1.876; 95% CI, 1.440–2.444; P-trend < 0.001). In contrast, no significant associations were identified in men across any model.

**Conclusion:**

Greater adherence to an unhealthy plant-based dietary pattern was associated with increased inflammation and adiposity among Korean women. While sensitivity analyses consistently supported these findings, the cross-sectional design means these results indicate associations, not causal relationships.

## 1 Introduction

Obesity is recognized as a critical global public health issue and a significant risk factor for various chronic diseases, including cardiovascular disease, type 2 diabetes, and several cancers (1, 2). According to the World Health Organization (WHO), the global prevalence of obesity among adults has more than doubled since 1990 and continues to rise steadily (2). Similar trends have been observed in East Asian countries, including Korea, where national statistics indicate a continuous increase in obesity prevalence among both men and women over the past decade (3). These trends not only reflect weight gain but also contribute to metabolic abnormalities, diminished quality of life, premature mortality, and escalating healthcare costs, imposing substantial social and economic burdens (4).

Recent years have witnessed a global transition in dietary behaviors, characterized by an increased consumption of restaurant foods, food-delivery meals, and fast foods, raising significant public health concerns (5, 6). Furthermore, restrictions on outdoor activities during the COVID-19 pandemic, which emerged in December 2019, have further accelerated the intake of processed and energy-dense foods (7). The excessive consumption of ultra-processed foods has been implicated as a major risk factor for obesity, hypertension, and hypertriglyceridemia (8). As a consequence of these dietary shifts, metabolic syndrome, traditionally more prevalent among middle-aged and older adults, has increasingly been reported in young adults aged 20–39 years (9). Rising rates of diabetes in younger populations, along with dyslipidemia and fatty liver disease, have become more common (10, 11). These phenomena are closely linked to modifications in dietary habits, suggesting that maintaining an overall healthy diet is critical for preventing and managing chronic diseases, even later in life (12–17).

Obesity, a condition intricately linked to metabolic disorders, is significantly associated with chronic low-grade inflammation, which is driven by the abnormal expansion and metabolic dysfunction of adipose tissue. The activation of macrophages and other immune cells within adipose tissue promotes the secretion of pro-inflammatory cytokines, such as interleukin-6 (IL-6) and tumor necrosis factor-alpha (TNF-*α*). These cytokines exacerbate systemic inflammation and facilitate the development of cardiovascular and metabolic diseases (18). Among inflammatory biomarkers, high-sensitivity C-reactive protein (hsCRP) has emerged as a prominent indicator for assessing cardiovascular risk and metabolic abnormalities (19, 20). According to the guidelines established by the Centers for Disease Control and Prevention and the American Heart Association (CDC/AHA), hsCRP levels are classified as follows: <1 mg/L (low risk), 1–3 mg/L (intermediate risk), and ≥3 mg/L (high risk). Elevated hsCRP levels are strongly associated with obesity, metabolic syndrome, and cardiovascular disease risk, rendering it a critical predictive biomarker in clinical and epidemiological studies (19).

The development of obesity and inflammation is influenced by an intricate interplay among physical activity, lifestyle factors, and comorbid conditions, with diet serving as a modifiable and particularly significant determinant. Dietary pattern approaches, such as the Mediterranean diet and the Dietary Approaches to Stop Hypertension (DASH), have been found to exert beneficial effects on weight reduction, metabolic health, and inflammation (21, 22).

In line with these dietary approaches, Satija et al. (23) introduced the Plant-based Diet Index (PDI), which includes both the healthy PDI (hPDI) and unhealthy PDI (uPDI), as tools for evaluating the quality of plant-based dietary patterns. The hPDI assigns higher scores for greater consumption of whole grains, vegetables, fruits, legumes, and nuts, while the uPDI assigns higher scores for the intake of refined grains, potatoes, and sugar-sweetened beverages—foods associated with an elevated risk of metabolic diseases (23). Previous studies indicate that closer adherence to the PDI or hPDI is linked to favorable outcomes regarding obesity and metabolic biomarkers, whereas stronger adherence to the uPDI is associated with poorer metabolic profiles and increased disease risk (23–27). Compared with the hPDI, the PDI reflects overall plant food intake and typically exhibits weaker associations (24), underscoring the importance of evaluating both the quantity and quality of plant-based foods.

However, most studies on plant-based dietary indices have focused on Western populations, whose dietary habits and major food sources differ substantially from those of East Asian populations (23–27). Traditional Korean diets feature high consumption of rice, vegetables, and fermented foods (28, 29), but have recently become increasingly Westernized (7, 8, 30, 31). Consequently, the associations of the uPDI with obesity- and inflammation-related markers in Korean adults may differ from those observed in Western populations.

Therefore, this study used nationally representative data from the 2022–2023 Korea National Health and Nutrition Examination Survey (KNHANES) to investigate the associations between the uPDI and obesity-related markers, including high-sensitivity C-reactive protein (hsCRP) and body mass index (BMI), among Korean adults. By examining these associations in an East Asian population with unique dietary characteristics, this study aimed to provide evidence regarding the relationship between plant-based diet quality and inflammation- and obesity-related markers.

## 2 Materials and methods

### 2.1 Study population and data source

This study used data from the 9th KNHANES (2022–2023), a nationally representative survey designed to assess the health, nutritional status, and lifestyle of the Korean population through a stratified, multistage cluster sampling design. A total of 13,194 individuals (5,885 men and 7,309 women) participated in 9th KNHANES. Among them, 7,727 adults aged 19–64 years were initially eligible for inclusion. Participants were sequentially excluded if they reported implausible total energy intakes (<500 or >5,000 kcal/day; *n* = 436), were underweight (BMI < 18.5 kg/m^2^; n = 401), or had missing hsCRP measurements (*n* = 1,280). After applying these exclusion criteria, 5,610 participants (2,607 men and 3,003 women) were included in the final analysis. All participants were interviewed by trained survey staff using standardized questionnaires, and anthropometric measurements and laboratory assessments were conducted in accordance with established protocols.

### 2.2 Dietary assessment and calculation of the uPDI

Dietary intake was assessed via a single 24-h dietary recall. Reported food items were categorized into 19 predefined food groups. Eighteen of these food groups were based on the original plant-based diet index framework proposed by Satija et al. (23), with an additional “salty foods” group included to reflect characteristics of the Korean diet. These groups were subsequently classified as healthy plant, unhealthy plant, or animal foods, consistent with the original scoring scheme. Daily intake for each food group was calculated and adjusted for total energy intake (g/1,000 kcal). Participants were ranked into quintiles for each food group based on their energy-adjusted intake, and quintile scores from 1 to 5 were assigned following the original scoring method. For the uPDI, higher consumption of unhealthy plant foods received positive scores (5 for the highest quintile, 1 for the lowest), while healthy plant and animal food groups were reverse-scored (1 for the highest, 5 for the lowest). Individual uPDI scores, ranging from 19 to 95, were calculated by summing the scores across all 19 food groups, with higher scores indicating greater adherence to an unhealthy plant-based dietary pattern (32). Supplementary Table S1 details the classification of individual food items into the 19 predefined food groups.

### 2.3 Outcome variables

Serum hsCRP concentrations were measured using an immunoturbidimetric assay (Cobas, Roche Diagnostics, Mannheim, Germany) per the standardized KNHANES laboratory protocol. Following CDC/AHA recommendations, hsCRP levels were categorized as low risk (<1 mg/L), intermediate risk (1–3 mg/L), and high risk (≥3 mg/L). Participants with hsCRP concentrations ≥3 mg/L were classified as having elevated systemic inflammation (19). BMI was calculated as weight (kg) divided by height squared (m^2^) and classified according to WHO Western Pacific Region criteria for Asian populations: normal weight (18.5–24.9 kg/m^2^) and obesity (≥25.0 kg/m^2^) (33).

### 2.4 Covariates

To minimize potential confounding, age and total energy intake were incorporated as essential adjustment variables. Additional covariates, including education level, household income, smoking status, alcohol consumption, physical activity, hsCRP, and BMI, were selected *a priori* based on existing literature and biological plausibility. Menopausal status was also considered in analyses involving women. Educational attainment was classified into four levels: ≤ elementary school, middle school, high school, and ≥ college. Household income was categorized into quartiles (lowest, lower-middle, upper-middle, and highest) based on equivalized household income. Smoking status was categorized into quintiles (low / Middle-low / Middle / Middle-high / High). Alcohol consumption was indicated as “yes” for participants who consumed alcohol at least once per month during the preceding year and “no” otherwise. Physical activity was dichotomized according to aerobic activity guidelines: participants engaging in ≥150 min per week of moderate-intensity activity, ≥75 min per week of vigorous-intensity activity, or an equivalent combination were deemed physically active; all others were classified as inactive. Among women, menopausal status was self-reported, with those experiencing natural or surgical menopause indicating “yes” and those currently menstruating, pregnant, breastfeeding, or reporting “unknown” indicating “no”.

### 2.5 Statistical analysis

All statistical analyses accounted for the complex sampling design of KNHANES by incorporating sampling weights, stratification, and clustering. General characteristics and dietary intakes were summarized according to sex and uPDI quartiles. Continuous variables are presented as weighted means with standard errors (SEs), whereas categorical variables are presented as weighted percentages. Differences across uPDI quartiles were evaluated using complex sample linear regression (PROC SURVEYREG) for continuous variables and complex sample logistic regression (PROC SURVEYLOGISTIC) for categorical variables. Linear trends across quartiles were assessed using P for trend. We evaluated associations between the uPDI and health outcomes using multivariable linear and logistic regression models. hsCRP and BMI were analyzed as continuous outcome variables in linear regression models, while elevated hsCRP (≥3 mg/L) and obesity (BMI ≥ 25 kg/m^2^) were analyzed as binary outcome variables in logistic regression models. Model 1 was unadjusted, while Model 2 was adjusted for age, total energy intake, education level, household income, smoking status, alcohol consumption, physical activity, and menopausal status in women. We conducted additional models that mutually adjusted for hsCRP (when BMI was the outcome) or BMI (when hsCRP was the outcome) as sensitivity analyses to evaluate the robustness of the observed associations. Considering the right-skewed distribution of hsCRP, we used log-transformed hsCRP values in linear regression analyses and additionally estimated geometric mean ratios (GMRs) and corresponding percent differences to facilitate interpretation on the original scale. All statistical analyses were performed using SAS version 9.4 (SAS Institute, Cary, NC, USA), and two-sided *p* values < 0.05 were considered statistically significant.

## 3 Results

### 3.1 General characteristics of study participants

The sociodemographic and health-related characteristics of the study population (*n* = 5,610) according to sex and uPDI quartiles are presented in Table 1. Most participants—90.8% of men and 91.0% of women—had normal hsCRP levels (<3 mg/L), while 9.2% of men and 9.0% of women exhibited elevated levels (≥3 mg/L). Regarding BMI, 47.4% of men had a normal BMI, compared to 67.1% of women. Consequently, 52.6% of men and 32.9% of women were classified as obese. Across uPDI quartiles, higher scores correlated with younger age in women (P-trend < 0.001) but not in men (P-trend = 0.574). Higher uPDI scores were also associated with lower household income and educational attainment in both sexes (P-trend < 0.001 for both). Similarly, higher uPDI scores in both men and women corresponded to lower physical activity levels (men: P-trend = 0.029; women: P-trend < 0.001) and a higher prevalence of current smoking (men: P-trend < 0.001; women: P-trend = 0.016). Increased alcohol consumption was observed with higher uPDI scores among women (P-trend = 0.002), but not in men (P-trend = 0.891). Menopausal status also varied significantly across uPDI quartiles among women (P-trend = 0.005).

**Table 1:** Participants’ general characteristics according to quartiles of the unhealthy plant-based diet index (uPDI).

Variables	Men					Women				
	Q1^a^ (*n* = 689)	Q2 (*n* = 691)	Q3 (*n* = 601)	Q4 (*n* = 626)	P-trend^b^	Q1 (*n* = 830)	Q2 (*n* = 771)	Q3 (*n* = 687)	Q4 (*n* = 715)	P-trend
Age (years)	43.3 ± 0.5^**b**^	42.0 ± 0.5	42.3 ± 0.5	43.7 ± 0.6	0.574	46.4 ± 0.5	44.4 ± 0.6	44.0 ± 0.6	43.4 ± 0.5	**<0.001**
Household income level (*n*, %)					**<0.001**					**<0.001**
Low	19(3.0)	30(3.9)	25(4.1)	69(9.7)		45(4.9)	38(5.5)	43(4.9)	67(7.3)	
Middle-low	70(10.5)	67(8.9)	73(11.4)	96(13.5)		120(13.2)	106(13.0)	121(16.6)	127(17.1)	
Middle	116(16.7)	144(20.5)	139(23.8)	155(24.2)		183(21.4)	169(20.7)	174(25.0)	180(25.3)	
Middle-high	219(32.4)	201(29.4)	173(28.8)	147(25.4)		209(27.0)	223(28.2)	180(27.1)	198(28.8)	
High	264(37.4)	249(37.3)	189(31.9)	158(27.2)		272(33.5)	233(32.6)	169(26.4)	143(21.5)	
Education level (*n*, %)					**<0.001**					**<0.001**
≤Elementary school	13(1.2)	6(0.7)	19(2.1)	29(3.5)		28(2.3)	42(3.5)	35(4.3)	60(6.4)	
Middle school graduate	20(2.3)	25(3.1)	26(2.8)	50(6.8)		42(4.0)	51(5.5)	54(6.6)	54(6.1)	
High school graduate	184(27.6)	234(33.2)	240(40.8)	274(43.7)		311(37.7)	282(38.0)	280(40.4)	302(44.0)	
≥College or university	469(68.9)	419(63.0)	312(54.3)	262(46.0)		439(56.0)	392(53.0)	314(48.7)	295(43.5)	
Physical activity (*n*, %)					**0.029**					**<0.001**
Yes	365(55.9)	375(59.6)	290(54.7)	280(50.2)		420(53.4)	368(51.7)	308(49.6)	290(44.1)	
No	291(44.1)	271(40.4)	269(45.3)	299(49.8)		377(46.6)	373(48.3)	336(50.4)	393(55.9)	
Smoking status (*n*, %)					**<0.001**					**0.016**
Yes	155(21.8)	229(32.6)	221(36.0)	293(45.9)		26(3.7)	37(5.1)	39(5.6)	51(7.1)	
No	532(78.2)	457(67.4)	378(64.0)	329(54.1)		799(96.3)	732(94.9)	646(94.4)	663(92.9)	
Alcohol consumption (*n*, %)					0.891					**0.002**
Yes	457(66.3)	501(73.6)	439(71.3)	429(67.2)		342(42.4)	378(51.0)	354(53.7)	349(51.0)	
No	230(33.7)	186(26.4)	161(28.7)	193(32.8)		483(57.6)	391(49.0)	331(46.3)	365(49.0)	
Menopausal status (*n*, %)										**0.005**
Yes	—	—	—	—	—	329(39.2)	289(35.0)	229(33.7)	240(31.3)	
No						409(60.8)	412(65.0)	375(66.3)	401(68.7)	
Energy intake (kcal)^c^	2233.4 ± 32.2	2197.6 ± 32.0	2195.6 ± 34.7	2013.4 ± 37.0	**<0.001**	1688.6 ± 23.1	1624.0 ± 23.0	1600.4 ± 27.9	1510.5 ± 28.2	0.172
hsCRP, mg/L	1.5 ± 0.2	1.2 ± 0.1	1.2 ± 0.1	1.6 ± 0.2	0.136	0.9 ± 0.1	1.3 ± 0.2	1.3 ± 0.1	1.6 ± 0.1	**<0.001**
BMI, kg/m^2^	25.8 ± 0.2	25.7 ± 0.2	25.6 ± 0.2	25.7 ± 0.2	0.470	23.5 ± 0.1	24.1 ± 0.2	24.2 ± 0.2	24.7 ± 0.2	**<0.001**

a The quartile of unhealthy plant-based diet index (uPDI) based on sex-specific distribution.

b P-trend was tested across uPDI quartiles, treating quartiles as ordinal. Survey-weighted linear regression (PROC SURVEYREG) was used for continuous variables, and logistic regression models (PROC SURVEYLOGISTIC) were applied for categorical variables, accounting for the complex survey design of KNHANES.

c Participants with implausible total energy intake (<500 kcal/day or >5,000 kcal/day) were excluded from the analyses.

*hsCRP, high-sensitivity C-reactive protein (mg/L); BMI, body mass index (kg/m^2^).

*Values are presented as mean ± standard error (SE) for continuous variables and as number (weighted %) for categorical variables.

*For hsCRP, 90.8% of men and 91.0% of women had normal levels (< 3 mg/L), whereas 9.2% of men and 9.0% of women exhibited elevated hsCRP levels (≥ 3 mg/L). After excluding underweight participants (BMI < 18.5 kg/m^2^), 47.4% of men and 67.1% of women had normal BMI (< 25 kg/m^2^), while 52.6% of men and 32.9% of women were obesity (BMI ≥ 25 kg/m^2^).*Bold values indicate statistical significance at *P*-trend < 0.05.

### 3.2 Daily food and nutrient intakes

Table 2 details daily food and nutrient intakes by uPDI quartile. Daily food intake varied across uPDI quartiles. Grain intake rose significantly with increasing uPDI scores in both men and women (both P-trend < 0.001). In contrast, consumption of legumes, nuts, vegetables, mushrooms, fruits, eggs, fish and shellfish, and milk and dairy products significantly decreased across increasing uPDI quartiles in both sexes. Meat intake, however, exhibited no significant trend. Neither beverage nor alcoholic beverage intake displayed significant trends across uPDI quartiles in either sex. Similar patterns emerged for nutrient intakes. As uPDI quartiles increased, intakes of protein; total, saturated, monounsaturated, and polyunsaturated fats; cholesterol; dietary fiber; calcium; phosphorus; potassium; magnesium; iron; zinc; vitamins A, D, E, B1, B2, B3, and folate; *β*-carotene; and vitamin C all decreased significantly (all P-trend < 0.001). Conversely, carbohydrate intake significantly increased in both sexes (both P-trend < 0.001). Sodium intake significantly increased exclusively among women (P-trend = 0.040), with no significant trend observed among men (P-trend = 0.068).

**Table 2:** Daily food and nutrient intakes of participants according to quartiles of the unhealthy plant-based diet index (uPDI).

Variables	Men					Women				
	Q1^a^ (*n* = 689)	Q2 (*n* = 691)	Q3 (*n* = 601)	Q4 (*n* = 626)	P-trend^b^	Q1 (*n* = 830)	Q2 (*n* = 771)	Q3 (*n* = 687)	Q4 (*n* = 715)	P-trend
Food intakes^c^										
Cereal and cereal products (g/day)	63.24 ± 1.67	74.51 ± 2.24	78.44 ± 2.18	94.04 ± 2.77	**<0.001**	76.40 ± 1.87	97.66 ± 3.20	105.67 ± 2.87	132.57 ± 3.55	**<0.001**
Potatoes and starchy products (g/day)	6.44 ± 0.75	6.94 ± 0.73	7.74 ± 1.00	8.21 ± 1.18	**0.021**	13.50 ± 1.06	12.46 ± 1.06	16.34 ± 1.53	16.99 ± 2.16	0.220
Sugar and sugary products (g/day)	2.18 ± 0.13	2.55 ± 0.27	2.15 ± 0.18	2.47 ± 0.34	0.486	3.32 ± 0.22	3.86 ± 0.25	4.22 ± 0.33	4.50 ± 0.35	**0.009**
Beans and bean products (g/day)	17.04 ± 1.43	12.17 ± 1.15	13.95 ± 2.67	6.29 ± 0.71	**<0.001**	28.59 ± 2.55	21.30 ± 2.41	18.11 ± 1.85	14.13 ± 1.67	**0.003**
Nuts and seeds (g/day)	1.83 ± 0.17	1.31 ± 0.19	1.40 ± 0.28	1.00 ± 0.20	**<0.001**	3.38 ± 0.36	3.64 ± 1.03	2.00 ± 0.27	3.40 ± 1.68	**<0.001**
Vegetables (g/day)	83.40 ± 2.79	78.13 ± 2.59	70.07 ± 2.41	79.79 ± 3.37	**<0.001**	120.68 ± 4.11	124.74 ± 6.46	117.46 ± 5.15	114.29 ± 5.25	**0.002**
Mushrooms (g/day)	2.44 ± 0.36	1.44 ± 0.15	1.07 ± 0.14	0.79 ± 0.15	**0.017**	3.67 ± 0.79	2.38 ± 0.42	3.12 ± 0.56	3.13 ± 0.48	**0.034**
Fruit (g/day)	38.02 ± 2.24	25.79 ± 2.27	20.46 ± 2.35	17.14 ± 1.95	**0.018**	81.17 ± 3.66	71.32 ± 4.76	58.71 ± 4.63	55.71 ± 6.06	**0.032**
Meat and meat products (g/day)	36.95 ± 1.40	35.45 ± 1.38	37.83 ± 1.83	33.16 ± 2.05	0.794	39.15 ± 1.62	39.90 ± 1.92	39.63 ± 2.11	35.37 ± 2.00	0.921
Eggs and egg products (g/day)	14.45 ± 0.81	10.57 ± 0.61	9.21 ± 0.78	7.00 ± 0.82	**<0.001**	23.86 ± 1.12	18.55 ± 1.22	15.99 ± 1.29	11.28 ± 0.89	**<0.001**
Fish and shellfish (g/day)	20.57 ± 1.39	15.80 ± 1.18	12.86 ± 1.26	8.40 ± 0.83	**<0.001**	23.70 ± 1.79	20.48 ± 1.38	19.14 ± 1.52	16.84 ± 3.21	**<0.001**
Seaweed (g/day)	1.30 ± 0.23	0.87 ± 0.12	1.11 ± 0.23	1.14 ± 0.15	**0.002**	1.84 ± 0.20	1.95 ± 0.28	1.65 ± 0.29	1.30 ± 0.15	0.918
Milk and dairy products (g/day)	29.66 ± 1.82	22.81 ± 1.88	17.08 ± 1.95	12.87 ± 2.05	**0.013**	62.90 ± 3.20	45.91 ± 3.11	39.04 ± 3.40	31.41 ± 3.34	**0.034**
Plant-based oils and fats (g/day)	2.49 ± 0.10	2.06 ± 0.09	1.70 ± 0.11	1.03 ± 0.08	**<0.001**	3.39 ± 0.13	2.82 ± 0.17	2.38 ± 0.13	1.44 ± 0.09	**<0.001**
Animal-based oils and fats (g/day)	0.12 ± 0.02	0.13 ± 0.03	0.11 ± 0.03	0.06 ± 0.02	0.242	0.30 ± 0.05	0.14 ± 0.03	0.10 ± 0.02	0.10 ± 0.03	0.295
Beverages (g/day)	106.71 ± 7.17	94.86 ± 5.72	82.15 ± 5.72	93.58 ± 7.67	0.195	151.92 ± 10.53	166.01 ± 16.98	139.60 ± 11.53	149.56 ± 11.82	0.121
Alcoholic beverages (g/day)	17.13 ± 2.26	23.89 ± 2.12	23.70 ± 2.48	27.22 ± 3.20	0.788	9.50 ± 1.77	16.36 ± 2.74	19.44 ± 3.31	25.35 ± 3.64	0.796
Seasonings (g/day)	12.07 ± 0.41	11.83 ± 0.48	12.83 ± 1.46	11.44 ± 0.93	**<0.001**	14.03 ± 0.54	14.76 ± 0.66	15.17 ± 0.68	15.43 ± 1.04	0.539
Processed foods (g/day)	0.45 ± 0.13	0.18 ± 0.06	0.19 ± 0.06	0.14 ± 0.08	0.091	0.73 ± 0.23	0.43 ± 0.11	0.61 ± 0.25	0.34 ± 0.12	0.235
Nutrient intakes^c^										
Protein (g)	43.27 ± 0.39	39.76 ± 0.40	38.17 ± 0.46	33.30 ± 0.44	**<0.001**	43.11 ± 0.43	39.68 ± 0.42	37.92 ± 0.43	33.63 ± 0.39	**<0.001**
Fat (g)	31.23 ± 0.41	27.51 ± 0.39	26.58 ± 0.45	23.14 ± 0.43	**<0.001**	32.51 ± 0.35	29.44 ± 0.43	26.71 ± 0.41	24.10 ± 0.50	**<0.001**
SFA (g)	9.25 ± 0.15	8.52 ± 0.16	8.49 ± 0.19	7.87 ± 0.19	**<0.001**	9.94 ± 0.16	9.34 ± 0.18	8.53 ± 0.18	8.29 ± 0.22	**<0.001**
MUFA (g)	10.22 ± 0.17	9.04 ± 0.16	8.88 ± 0.19	7.85 ± 0.18	**<0.001**	10.36 ± 0.14	9.47 ± 0.17	8.69 ± 0.16	7.86 ± 0.20	**<0.001**
PUFA (g)	8.60 ± 0.16	7.18 ± 0.13	6.50 ± 0.14	5.19 ± 0.11	**<0.001**	8.86 ± 0.15	7.58 ± 0.15	6.76 ± 0.14	5.51 ± 0.13	**<0.001**
Omega-3 fatty acids (g)	1.21 ± 0.03	0.98 ± 0.03	0.89 ± 0.03	0.68 ± 0.02	**<0.001**	1.38 ± 0.04	1.13 ± 0.03	1.06 ± 0.05	0.74 ± 0.03	**<0.001**
Omega-6 fatty acids (g)	7.37 ± 0.14	6.17 ± 0.11	5.59 ± 0.12	4.49 ± 0.09	**<0.001**	7.46 ± 0.13	6.42 ± 0.13	5.68 ± 0.11	4.73 ± 0.11	**<0.001**
Cholesterol (mg)	184.28 ± 4.08	151.48 ± 3.51	138.96 ± 5.12	99.07 ± 3.41	**<0.001**	194.42 ± 3.78	162.46 ± 3.96	146.16 ± 4.60	110.23 ± 3.44	**<0.001**
Carbohydrates (g)	128.55 ± 1.09	133.68 ± 1.33	138.63 ± 1.45	149.27 ± 1.51	**<0.001**	131.94 ± 1.09	140.01 ± 1.23	145.93 ± 1.37	154.83 ± 1.53	**<0.001**
Dietary fiber (g)	13.00 ± 0.22	11.94 ± 0.20	11.54 ± 0.21	11.72 ± 0.20	**<0.001**	14.93 ± 0.23	14.15 ± 0.24	13.42 ± 0.27	13.08 ± 0.24	**<0.001**
Sugars (g)	30.21 ± 0.66	27.58 ± 0.65	26.73 ± 0.78	27.46 ± 0.81	**<0.001**	36.64 ± 0.64	35.40 ± 0.81	33.86 ± 0.79	34.23 ± 0.92	**<0.001**
Calcium (mg)	285.00 ± 5.14	248.42 ± 4.76	235.41 ± 5.39	233.58 ± 5.31	**<0.001**	357.80 ± 6.90	300.65 ± 5.79	279.56 ± 6.16	256.73 ± 5.35	**<0.001**
Phosphorus (mg)	620.76 ± 5.11	559.30 ± 4.89	535.59 ± 6.18	479.27 ± 5.47	**<0.001**	667.09 ± 5.96	606.69 ± 5.28	573.15 ± 6.03	513.31 ± 5.21	**<0.001**
Sodium (mg)	1869.23 ± 28.19	1837.00 ± 31.31	1803.11 ± 29.20	1841.78 ± 35.40	0.068	1676.90 ± 29.87	1725.37 ± 28.11	1742.77 ± 29.15	1796.49 ± 36.35	**0.040**
Potassium (mg)	1556.18 ± 17.89	1373.21 ± 16.96	1288.38 ± 16.92	1199.37 ± 17.73	**<0.001**	1750.56 ± 19.76	1626.45 ± 24.51	1522.45 ± 23.98	1360.54 ± 21.43	**<0.001**
Magnesium (mg)	168.69 ± 2.17	153.50 ± 1.98	148.15 ± 2.30	140.84 ± 1.97	**<0.001**	186.37 ± 2.55	170.42 ± 2.37	164.81 ± 2.43	151.97 ± 2.16	**<0.001**
Iron (mg)	5.45 ± 0.11	5.10 ± 0.11	5.14 ± 0.16	4.47 ± 0.20	**<0.001**	5.96 ± 0.11	5.45 ± 0.11	5.43 ± 0.15	4.60 ± 0.11	**<0.001**
Zinc (mg)	5.81 ± 0.07	5.52 ± 0.07	5.40 ± 0.07	4.83 ± 0.08	**<0.001**	5.82 ± 0.07	5.62 ± 0.08	5.43 ± 0.07	4.92 ± 0.06	**<0.001**
Vitamin A (μg RAE)	235.98 ± 8.87	205.30 ± 6.50	176.15 ± 6.01	144.60 ± 5.56	**<0.001**	282.68 ± 7.26	255.78 ± 8.66	241.10 ± 9.56	186.35 ± 6.83	**<0.001**
Vitamin D (μg)	2.23 ± 0.15	1.61 ± 0.10	1.45 ± 0.11	1.00 ± 0.08	**<0.001**	2.07 ± 0.10	1.87 ± 0.11	1.69 ± 0.12	1.18 ± 0.09	**<0.001**
Vitamin E (mg α-TE)	4.28 ± 0.07	3.68 ± 0.05	3.57 ± 0.07	3.35 ± 0.05	**<0.001**	4.62 ± 0.07	4.08 ± 0.06	3.93 ± 0.07	3.51 ± 0.06	**<0.001**
β-carotene (μg)	1503.67 ± 52.68	1395.29 ± 47.01	1223.30 ± 44.77	1036.17 ± 37.33	**<0.001**	1889.28 ± 72.49	1778.65 ± 89.41	1678.03 ± 81.79	1358.10 ± 62.01	**<0.001**
Retinol (μg)	107.63 ± 7.16	87.41 ± 5.61	72.07 ± 4.73	57.37 ± 5.17	**<0.001**	122.38 ± 4.56	105.49 ± 4.95	99.83 ± 7.44	72.29 ± 5.10	**<0.001**
Vitamin B1 (mg)	0.67 ± 0.01	0.63 ± 0.01	0.61 ± 0.01	0.55 ± 0.01	**<0.001**	0.65 ± 0.01	0.63 ± 0.01	0.61 ± 0.01	0.58 ± 0.01	**<0.001**
Vitamin B2 (mg)	0.93 ± 0.01	0.88 ± 0.02	0.85 ± 0.02	0.86 ± 0.02	**<0.001**	1.02 ± 0.01	0.93 ± 0.02	0.90 ± 0.01	0.87 ± 0.02	**<0.001**
Vitamin B3 (mg)	7.49 ± 0.11	6.97 ± 0.14	6.74 ± 0.15	6.00 ± 0.13	**<0.001**	7.48 ± 0.13	7.11 ± 0.13	6.81 ± 0.14	6.11 ± 0.13	**<0.001**
Folate (μg DFE)	171.04 ± 2.89	157.85 ± 2.96	146.68 ± 2.83	142.94 ± 2.45	**<0.001**	197.22 ± 3.42	182.61 ± 3.28	176.93 ± 3.68	161.11 ± 3.11	**<0.001**
Vitamin C (mg)	38.99 ± 1.58	36.35 ± 2.76	26.89 ± 1.44	31.42 ± 3.60	**<0.001**	46.21 ± 1.75	43.66 ± 2.08	44.76 ± 2.60	41.78 ± 3.41	**<0.001**

a Quartiles of the unhealthy plant-based diet index (uPDI) were generated separately for men and women (Q1: lowest quartile, Q2: second quartile, Q3: third quartile, Q4: highest quartile).

b P-trend were derived by treating uPDI quartiles as an ordinal variable (1–4) in survey-weighted linear regression models. All food group and nutrient intake variables were natural log-transformed prior to trend analysis due to skewed distributions; zero values were excluded from log transformation and treated as missing.

c All food and nutrient intake variables were expressed as energy-adjusted intakes (per 1,000 kcal) using the energy-density method.

*Values are presented as mean ± standard error (SE) and were estimated using PROC SURVEYMEANS, accounting for the complex sampling design of the KNHANES.

*Participants with implausible total energy intake (<500 kcal/day or >5,000 kcal/day) were excluded from the analyses.*Bold values indicate statistical significance at *P*-trend < 0.05.

### 3.3 Associations of the uPDI with hsCRP and BMI

Table 3 illustrates the sex-specific associations of uPDI with hsCRP and BMI. For men, the uPDI did not demonstrate a significant association with either hsCRP or BMI in crude (Model 1) or adjusted (Model 2) models. In contrast, women with higher uPDI scores exhibited significantly higher hsCRP and BMI in both crude and adjusted analyses (all *p* < 0.001). In the fully adjusted model (Model 2), each 1-unit increase in the uPDI score correlated with a 1.81% rise in hsCRP (GMR: 1.018; 95% CI, 1.012–1.025) and a 0.075 kg/m^2^ increase in BMI (*β* = 0.075, SE = 0.015). Sensitivity analyses, which further adjusted for BMI (Model 3) or hsCRP (Model 4), yielded comparable results. Among women, the uPDI–hsCRP association remained significant after additional BMI adjustment (*β* = 0.011, SE = 0.003, *p* < 0.001; GMR: 1.011; 95% CI, 1.005–1.016), and the uPDI–BMI association persisted after further hsCRP adjustment (*β* = 0.040, SE = 0.012, *p* = 0.001). No significant associations were observed among men during either sensitivity analysis.

**Table 3:** Associations of the unhealthy plant-based diet index (uPDI) with hsCRP and BMI among Korean adults aged 19–64 years, stratified by sex.

Variables	Men (*n* = 2,607)					Women (*n* = 3,003)				
	*β* (SE)	*p*-value	GMR	95% CI	% difference	*β*(SE)	*p*-value	GMR	95% CI	% difference
hsCRP										
Model 1^a^	0.006 (0.003)	0.089	1.005	0.999–1.012	+0.55% (−0.08–1.18%)	0.018(0.003)	**<0.001**	1.019	1.012–1.025	+1.85% (1.23–2.47%)
Model 2^b^	0.001 (0.004)	0.757	1.001	0.994–1.008	+0.11% (−0.58–0.80%)	0.018 (0.003)	**<0.001**	1.018	1.012–1.025	+1.81% (1.15–2.46%)
BMI										
Model 1	−0.017(0.014)	0.226				0.079(0.013)	**<0.001**			
Model 2	−0.021(0.014)	0.156				0.075(0.015)	**<0.001**			
hsCRP (adj. For BMI)										
Model 3^c^	0.002 (0.003)	0.500	1.002	0.996–1.009	+0.23% (−0.44–0.90%)	0.011 (0.003)	**<0.001**	1.011	1.005–1.016	+1.07% (0.51–1.64%)
BMI (adj. For hsCRP)										
Model 4^d^	−0.022(0.014)	0.120				0.040(0.012)	**0.001**			

a Model 1: Crude model without adjustment.

b Model 2: Adjusted for age, total energy intake, education level, household income, smoking status, alcohol consumption, and physical activity; in women, additionally adjusted for menopausal status.

c Model 3: Additionally adjusted for BMI, based on Model 2 (hsCRP as the dependent variable).

d Model 4: Additionally adjusted for hsCRP, based on Model 2 (BMI as the dependent variable).

^*^β coefficients and standard errors (SE) were estimated using survey-weighted linear regression models accounting for the complex sampling design of KNHANES.

^*^hsCRP values were log-transformed (natural log) for analysis. Geometric mean ratio (GMR) was calculated as exp(*β*), representing the multiplicative change in hsCRP levels (mg/L) per 1-unit increase in uPDI_ND score. ^*^Percent difference was calculated as (exp(*β*) − 1) × 100. GMR and percent difference are presented only for hsCRP models; BMI was analyzed on its original scale and *β* coefficients represent the change in BMI (kg/m^2^) per 1-unit increase in uPDI_ND score.*Bold values indicate statistical significance at *p*-trend < 0.05.

### 3.4 Associations of uPDI quartiles with elevated hsCRP and obesity by sex

Table 4 details the associations between uPDI quartiles and elevated hsCRP or obesity by sex. Mean uPDI scores significantly increased across quartiles for both men and women (both P-trend < 0.001). Among men, no significant associations were observed between uPDI quartiles and elevated hsCRP or obesity in either Model 1 or Model 2. After multivariable adjustment, no significant association was found between uPDI quartiles and elevated hsCRP (P-trend = 0.629) or obesity (P-trend = 0.125). For women, higher uPDI quartiles were linked to increased odds of elevated hsCRP. In Model 2, odds ratios (ORs) were significantly higher for Q2 (OR: 1.581; 95% confidence interval [CI], 1.020–2.451) and Q4 (OR: 2.043; 95% CI, 1.298–3.217) than for Q1, while the association for Q3 was positive, albeit not statistically significant (OR: 1.343; 95% CI, 0.845–2.135). Nevertheless, the overall trend across uPDI quartiles remained significant (P-trend = 0.006). Higher uPDI quartiles in women also correlated with increased odds of obesity. Following multivariable adjustment, the ORs were 1.537 (95% CI, 1.199–1.969), 1.644 (95% CI, 1.236–2.188), and 1.876 (95% CI, 1.440–2.444) for Q2, Q3, and Q4, respectively, compared with that for Q1, demonstrating a significantly positive trend across quartiles (P-trend < 0.001).

**Table 4:** ORs (95% CIs) of having elevated hsCRP (≥ 3 mg/L) or obesity (BMI ≥25kg/m2 ) by uPDI quartile.

Variables	Men					Women				
	Q1^a^ (*n* = 689)	Q2 (*n* = 691)	Q3 (*n* = 601)	Q4 (*n* = 626)	*P*-trend^b^	Q1 (*n* = 830)	Q2 (*n* = 771)	Q3 (*n* = 687)	Q4 (*n* = 715)	*P*-trend^d^
Dietary unhealthy plant-based diet index (uPDI) score	49.82 ± 0.12	55.47 ± 0.05	59.42 ± 0.04	65.09 ± 0.12	**<0.001**	47.73 ± 0.12	53.58 ± 0.04	57.37 ± 0.05	63.60 ± 0.13	**<0.001**
hsCRP										
Model 1^c^	1.000 (Reference)	1.171 (0.785–1.746)	0.858 (0.545–1.349)	1.144 (0.759–1.724)	0.880	1.000 (Reference)	1.570 (1.027–2.400)	1.510 (0.994–2.293)	2.217 (1.454–3.380)	**<0.001**
Model 2^d^	1.00 (Reference)	1.192 (0.789–1.800)	0.791 (0.493–1.270)	1.021 (0.660–1.580)	0.629	1.00 (Reference)	1.581 (1.020–2.451)	1.343 (0.845–2.135)	2.043 (1.298–3.217)	**0.006**
BMI										
Model 1	1.000 (Reference)	0.877 (0.711–1.083)	0.770 (0.605–0.982)	0.837 (0.660–1.060)	0.077	1.000 (Reference)	1.449 (1.163–1.805)	1.637 (1.272–2.107)	1.969 (1.562–2.481)	**<0.001**
Model 2	1.000 (Reference)	0.885 (0.710–1.103)	0.753 (0.588–0.965)	0.867 (0.676–1.112)	0.125	1.000 (Reference)	1.537 (1.199–1.969)	1.644 (1.236–2.188)	1.876 (1.440–2.444)	**<0.001**

a Quartiles of the unhealthy plant-based diet index (uPDI) were generated separately for men and women, based on the distribution of uPDI_ND scores.

b P-trend was derived from a separate survey-weighted logistic regression model treating uPDI quartiles as an ordinal variable (1–4).

c Model 1: Crude model without adjustment.

d Model 2: Adjusted for age, total energy intake, education level, household income, smoking status, alcohol consumption, and physical activity; in women, additionally adjusted for menopausal status.

*Odds ratios (ORs) and 95% confidence intervals (CIs) were estimated using survey-weighted logistic regression models (PROC SURVEYLOGISTIC), accounting for the complex sampling design of KNHANES.

*Elevated hsCRP was defined as hsCRP ≥ 3 mg/L; Obesity was defined as BMI ≥ 25.0 kg/m^2^.*Bold values indicate statistical significance at *P*-trend < 0.05.

## 4 Discussion

This study investigated sex-specific associations between the uPDI, hsCRP, and BMI using nationally representative data from the 2022–2023 KNHANES. Higher uPDI scores were linked to elevated hsCRP concentrations and BMI among women, despite no significant associations in men. These associations remained significant after multivariable adjustment and were consistently observed in sensitivity analyses, even with additional adjustment for BMI or hsCRP.

Several biological mechanisms may help explain the stronger associations observed in women. Women typically have a higher percentage of body fat than men, and their adipose tissue may be more metabolically active and responsive to inflammatory stimuli (34–36). Adipose tissue plays an important role in regulating glucose metabolism and insulin sensitivity (37). In obesity, expansion of adipose tissue promotes the secretion of pro-inflammatory cytokines, including IL-6 and TNF-*α*, which subsequently stimulate hepatic CRP production (38). Furthermore, clinical evidence indicates that premenopausal women exhibit greater insulin sensitivity than postmenopausal women and men (39), with a substantial increase in the prevalence of insulin resistance and metabolic syndrome occurring after menopause (40). These findings emphasize the regulatory role of estrogen in insulin responsiveness and its significance in adipose tissue development and function (41, 42). These biological differences may partly account for the stronger associations among uPDI, hsCRP, and BMI observed in women.

Behavioral factors may also contribute to these sex-specific findings. The rising prevalence of metabolic syndrome among young adults, especially women, has been linked to dietary behaviors and lifestyle patterns (43). Metabolic syndrome is closely associated with dietary factors, including overall eating habits (44). The increased consumption of delivery foods, fast foods, and ultra-processed foods (5, 6) has coincided with an escalation in early-onset metabolic disorders (9–11). Considering that the uPDI is characterized by a greater intake of refined grains, sweets, salty foods, and other less healthy plant-based or animal-derived foods, our findings suggest that these dietary components may be associated with abdominal obesity, elevated fasting glucose, and hypertriglyceridemia among women (45).

Previous studies have revealed that adherence to high-quality plant-based dietary patterns—such as the Mediterranean, DASH, Nordic, vegan, and vegetarian diets—is associated with weight reduction and a decreased likelihood of obesity-related chronic diseases (46, 47). In contrast, higher uPDI scores have been associated with higher Dietary Inflammatory Index (DII) scores, greater adiposity, and increased risks of cardiometabolic diseases in predominantly Western populations (48–51). Evidence from prior studies further indicates that stronger adherence to the hPDI correlates with decreased hsCRP levels, lower BMI, and reduced weight gain, while higher uPDI scores are positively associated with elevated hsCRP, IL-6, and TNF-*α* levels, along with increased BMI and obesity (52–55). Among Iranian women, higher hPDI scores have been associated with lower concentrations of hsCRP and transforming growth factor-beta, while the uPDI exhibited positive correlations with these inflammatory markers (52, 53). Comparable patterns emerged in the PATHWAYS study involving survivors of breast cancer, where the hPDI demonstrated inverse correlations with hsCRP, whereas the uPDI exhibited positive correlations (54).

Participants with higher uPDI scores also generally had poorer dietary quality. Specifically, women with higher uPDI scores consumed less protein, dietary fiber, vitamins, and minerals, while consuming more sugar-containing foods and sodium. These dietary characteristics suggest that higher uPDI scores reflect an overall diet of lower nutritional quality, partly explaining the observed associations with inflammatory and obesity-related markers.

### 4.1 Strengths of the study

1. National representativeness: Data were obtained from the KNHANES, a large nationally representative survey, ensuring the generalizability of results to the Korean adult population.
2. Sensitivity analyses: We conducted additional sensitivity analyses, mutually adjusting for hsCRP, and found consistent results, which reinforces the stability of the observed associations.
3. Sex-specific analysis: Our stratified analyses revealed sex-specific associations, offering insights that could inform future sex-tailored nutritional strategies.
4. Comprehensive covariate control: We accounted for various sociodemographic and lifestyle factors in our analyses, thereby minimizing the potential for confounding.
5. Focus on contemporary Korean data: This study is among the first to utilize recent KNHANES data that includes hsCRP, capturing current trends in dietary transition and Westernization in Korea.

### 4.2 Limitations

1. Cross-sectional design: As this was a cross-sectional study, we cannot establish causal relationships, nor can we rule out the possibility of reverse causation.
2. 24-h dietary recall: Dietary data were collected using a single 24-h recall. This method may not fully capture habitual dietary intake and might have led to non-differential exposure misclassification, potentially weakening the observed associations.
3. Potential selection bias: We excluded participants with missing hsCRP measurements. These excluded participants differed from those included in several baseline characteristics, such as age and BMI (Supplementary Table S2) . While necessary for our primary analyses, this exclusion might have affected the representativeness of our final analytic sample.
4. Cultural adaptation of the uPDI: Although we calculated the uPDI using its original scoring framework, the predefined food-group classification may not entirely capture the unique characteristics of the traditional Korean diet, including fermented foods, mixed grains, and mixed dishes.
5. Single hsCRP measurement: hsCRP levels were measured only once and might have been affected by transient infections or acute inflammatory conditions; therefore, future studies should incorporate repeated measurements or additional inflammatory markers (e.g., IL-6 and TNF-*α*, among others).
6. Unexplained sex-specific associations: We observed significant associations exclusively among women. As this was an observational study, we could not fully elucidate the biological and behavioral mechanisms underlying these sex-specific associations. Thus, future prospective studies should investigate them.

## 5 Conclusion

This study utilized nationally representative data from the 2022–2023 KNHANES to examine the associations of the uPDI with hsCRP and BMI among Korean adults. We found that higher uPDI scores were significantly associated with elevated hsCRP and obesity in women, and these associations remained consistent even after mutual adjustment for hsCRP and BMI. In contrast, we found no significant associations in men.

These findings suggest that unhealthy plant-based dietary patterns, characterized by a greater consumption of refined grains, sugar-rich foods, and other less healthy plant-based foods, may be linked to systemic inflammation and obesity-related outcomes. Therefore, when promoting plant-based diets, it is crucial to emphasize not only increasing plant food intake but also improving the quality of the plant-based foods consumed.

Owing to this study’s cross-sectional design, we cannot establish causal relationships. Future prospective and intervention studies are warranted to clarify the causality of these associations and determine whether the observed sex-specific associations can inform future dietary recommendations for Korean adults.

## Data Availability

Publicly available datasets were analyzed in this study. This data can be found here: https://knhanes.kdca.go.kr/knhanes/eng/main.do.

## References

[1] Hruby A Hu FB. The epidemiology of obesity: a big picture. PharmacoEconomics. (2015) 33:673–89. doi: 10.1007/s40273-014-0243-x, 25471927 PMC4859313

[2] World Health Organization. Obesity and overweight: fact sheet. (2021). Available online at: https://www.who.int/news-room/fact-sheets/detail/obesity-and-overweight (Accessed July 22, 2026).

[3] Korea Disease Control and Prevention Agency (KDCA). 2022 National Health Statistics: Korea National Health and Nutrition Examination Survey. Cheongju: Korea Disease Control and Prevention Agency. (2023).

[4] Swinburn BA Sacks G Hall KD McPherson K Finegood DT Moodie ML . The global obesity pandemic: shaped by global drivers and local environments. Lancet. (2011) 378:804–14. doi: 10.1016/S0140-6736(11)60813-1, 21872749

[5] Kant AK Whitley MI Graubard BI. Away from home meals: associations with biomarkers of chronic disease and dietary intake in American adults, NHANES 2005–2010. Int J Obes. (2015) 39:820–7. doi: 10.1038/ijo.2014.183, 25319744 PMC4400187

[6] Nugrahani MR. Health literacy and geography: examining environmental and socioeconomic influences on public health awareness. J Health Lit Qual Res. (2023) 3:46–56. doi: 10.61194/jhlqr.v3i1.544

[7] Abdelkawy K Elbarbry F El-Masry SM Zakaria AY Rodríguez-Pérez C El-Khodary NM. Changes in dietary habits during Covid-19 lockdown in Egypt: the Egyptian COVIDiet study. BMC Public Health. (2023) 23:15777. doi: 10.1186/s12889-023-15777-7, 37231373 PMC10209922

[8] Shim JS Ha KH Kim DJ Kim HC. Ultra-processed food consumption and obesity in Korean adults. Diabetes Metab J. (2023) 47:547–58. doi: 10.4093/dmj.2022.0026, 37095686 PMC10404531

[9] Kim HM Kang HJ Lee DH Jeong SM Joh HK. Association between breakfast frequency and metabolic syndrome among young adults in South Korea. Sci Rep. (2023) 13:16826. doi: 10.1038/s41598-023-43957-3, 37803107 PMC10558535

[10] Ko M Ha K. Association of delivered food consumption with dietary behaviors and obesity among young adults in Jeju. J Nutr Health. (2024) 57:336–48. doi: 10.4163/jnh.2024.57.3.336

[11] Kim JY Lee J Kim SG Kim NH. Recent glycemia is a major determinant of *β*-cell function in type 2 diabetes mellitus. Diabetes Metab J. (2024) 48:1135–46. doi: 10.4093/dmj.2023.0359, 38889769 PMC11621653

[12] Kim J Lee Y Won CW Kim MK Kye S Shim JS . Dietary patterns and frailty in older Korean adults: results from the Korean frailty and aging cohort study. Nutrients. (2021) 13:601. doi: 10.3390/nu13020601, 33673185 PMC7918700

[13] Oh C No J. The quality of a traditional dietary pattern in relation to metabolic syndrome in elderly south Koreans. J Obes Metab Syndr. (2018) 27:254–61. doi: 10.7570/jomes.2018.27.4.254, 31089571 PMC6513305

[14] Mao D Li G Liang M Wang S Ren X. Dietary patterns and multiple chronic diseases in older adults. Nutr Metab (Lond). (2024) 21:36. doi: 10.1186/s12986-024-00814-y, 38915027 PMC11194917

[15] Wang X Yan X Zhang J Pan S Li R Cheng L . Associations of healthy eating patterns with biological aging: National Health and nutrition examination survey (NHANES) 1999–2018. Nutr J. (2024) 23:112. doi: 10.1186/s12937-024-01017-0, 39342289 PMC11439248

[16] Kim J Lee Y Lee SY Kim YO Chung YS Park SB. Dietary patterns and functional disability in older Korean adults. Maturitas. (2013) 76:160–4. doi: 10.1016/j.maturitas.2013.07.011, 23953845

[17] Pengpid S Peltzer K. Multimorbidity in chronic conditions: public primary care patients in four greater Mekong countries. Int J Environ Res Public Health. (2017) 14:1019. doi: 10.3390/ijerph14091019, 28878150 PMC5615556

[18] Hotamisligil GS. Inflammation and metabolic disorders. Nature. (2006) 444:860–7. doi: 10.1038/nature05485, 17167474

[19] Pearson TA Mensah GA Alexander RW Anderson JL Cannon RO Criqui M . Markers of inflammation and cardiovascular disease: application to clinical and public health practice: a statement for healthcare professionals from the Centers for Disease Control and Prevention and the American Heart Association. Circulation. (2003) 107:499–511. doi: 10.1161/01.cir.0000052939.59093.45, 12551878

[20] Ridker PM. High-sensitivity C-reactive protein and cardiovascular risk: rationale for screening and primary prevention. Am J Cardiol. (2003) 92:17–22. doi: 10.1016/S0002-9149(03)00774-4, 12948872

[21] Valenzuela-Fuenzalida JJ Silva Bravo V Moyano Valarezo L Delgado Retamal MF Matta Leiva J Bruna-Mejías A . Effectiveness of DASH diet versus other diet modalities in patients with metabolic syndrome: a systematic review and meta-analysis. Nutrients. (2024) 16:3054. doi: 10.3390/nu16183054 39339654 PMC11434995

[22] Godos J Zappalà G Bernardini S Giambini I Bes-Rastrollo M Martinez-Gonzalez MA. Adherence to the Mediterranean diet is inversely associated with metabolic syndrome occurrence: a meta-analysis of observational studies. Int J Food Sci Nutr. (2017) 68:138–48. doi: 10.1080/09637486.2016.1221900, 27557591

[23] Satija A Bhupathiraju SN Rimm EB Spiegelman D Chiuve SE Borgi L . Plant-based dietary patterns and incidence of type 2 diabetes in US men and women: results from three prospective cohort studies. PLoS Med. (2016) 13:e1002039. doi: 10.1371/journal.pmed.1002039, 27299701 PMC4907448

[24] Wang YB Shivappa N Hébert JR Page AJ Gill TK Melaku YA. Association between dietary inflammatory index, dietary patterns, plant-based dietary index and the risk of obesity. Nutrients. (2021) 13:1536. doi: 10.3390/nu13051536, 34063221 PMC8147427

[25] Kharaty S Harrington JM Millar SR Perry IJ Phillips CM. Plant-based dietary indices and biomarkers of chronic low-grade inflammation: a cross-sectional analysis of adults in Ireland. Eur J Nutr. (2023) 62:3397–410. doi: 10.1007/s00394-023-03242-5, 37658860 PMC10611858

[26] Zhuang P Wang F Yao J Liu X Li Y Ao Y . Unhealthy plant-based diet is associated with a higher cardiovascular disease risk in patients with prediabetes and diabetes: a large-scale population-based study. BMC Med. (2024) 22:485. doi: 10.1186/s12916-024-03683-7, 39443972 PMC11515529

[27] Vajdi M Karimi A Zahiri Tousi A Hosseini B Nikniaz Z Abbasalizad Farhangi M. Association between plant-based diets and metabolic syndrome in obese adults from Iran: a cross-sectional study. BMC Endocr Disord. (2023) 23:109. doi: 10.1186/s12902-023-01358-7, 37193979 PMC10186771

[28] Song Y Joung H. A traditional Korean dietary pattern and metabolic syndrome abnormalities. Nutr Metab Cardiovasc Dis. (2012) 22:456–62. doi: 10.1016/j.numecd.2010.09.002, 21215606

[29] Kim SH Kim MS Lee MS Park YS Lee HJ Kang SA . Korean diet: characteristics and historical background. J Ethn Foods. (2016) 3:26–31. doi: 10.1016/j.jef.2016.03.002

[30] Shim JS Shim SY Cha HJ Kim J Kim HC. Association between ultra-processed food consumption and dietary intake and diet quality in Korean adults. J Acad Nutr Diet. (2022) 122:583–94. doi: 10.1016/j.jand.2021.07.012, 34463621

[31] Lee SJ Lee KW. Gender differences in ultra-processed food consumption and its association with obesity among Korean adults. Nutrients. (2025) 17:2027. doi: 10.3390/nu17122027, 40573138 PMC12196032

[32] Shin N Kim J. Association between different types of plant-based diet and dyslipidaemia in Korean adults. Br J Nutr. (2022) 128:542–8. doi: 10.1017/S0007114521003482, 34503592

[33] WHO Expert Consultation. Appropriate body-mass index for Asian populations and its implications for policy and intervention strategies. Lancet. (2004) 363:157–63. doi: 10.1016/S0140-6736(03)15268-3 14726171

[34] Blaak E. Gender differences in fat metabolism. Curr Opin Clin Nutr Metab Care. (2001) 4:499–502. doi: 10.1097/00075197-200111000-00006, 11706283

[35] Carpenter CL Yan E Chen S Hong K Arechiga A Kim WS . Body fat and body-mass index among a multiethnic sample of college-age men and women. J Obes. (2013) 2013:790654. doi: 10.1155/2013/790654, 23691288 PMC3649342

[36] Khera A Vega GL Das SR Ayers C McGuire DK Grundy SM . Sex differences in the relationship between C-reactive protein and body fat. J Clin Endocrinol Metab. (2009) 94:3251–8. doi: 10.1210/jc.2008-2406, 19567538 PMC2741708

[37] Rosen ED Spiegelman BM. Adipocytes as regulators of energy balance and glucose homeostasis. Nature. (2006) 444:847–53. doi: 10.1038/nature05483, 17167472 PMC3212857

[38] Weisberg SP McCann D Desai M Rosenbaum M Leibel RL Ferrante AW Jr. Obesity is associated with macrophage accumulation in adipose tissue. J Clin Invest. (2003) 112:1796–808. doi: 10.1172/JCI19246, 14679176 PMC296995

[39] Oya J Nakagami T Yamamoto Y Fukushima S Takeda M Endo Y . Effects of age on insulin resistance and secretion in subjects without diabetes. Intern Med. (2014) 53:941–7. doi: 10.2169/internalmedicine.53.1580, 24785884

[40] Pu D Tan R Yu Q Wu J. Metabolic syndrome in menopause and associated factors: a meta-analysis. Climacteric. (2017) 20:583–91. doi: 10.1080/13697137.2017.1386649, 29064321

[41] De Paoli M Zakharia A Werstuck GH. The role of estrogen in insulin resistance: a review of clinical and preclinical data. Am J Pathol. (2021) 191:1490–8. doi: 10.1016/j.ajpath.2021.05.011, 34102108

[42] Kuryłowicz A. Estrogens in adipose tissue physiology and obesity-related dysfunction. Biomedicine. (2023) 11:690. doi: 10.3390/biomedicines11030690, 36979669 PMC10045924

[43] Choi JH Woo HD Lee JH Kim J. Dietary patterns and risk for metabolic syndrome in Korean women: a cross-sectional study. Medicine (Baltimore). (2015) 94:e1424. doi: 10.1097/MD.0000000000001424, 26313795 PMC4602901

[44] Woo HD Shin A Kim J. Dietary patterns of Korean adults and the prevalence of metabolic syndrome: a cross-sectional study. PLoS One. (2014) 9:e111593. doi: 10.1371/journal.pone.0111593, 25365577 PMC4218781

[45] Kim H Lee K Rebholz CM Kim J. Association between unhealthy plant-based diets and the metabolic syndrome in adult men and women: a population-based study in South Korea. Br J Nutr. (2021) 125:577–90. doi: 10.1017/S0007114520002895, 32713361

[46] Kim H Caulfield LE Garcia-Larsen V Steffen LM Coresh J Rebholz CM. Plant-based diets are associated with a lower risk of incident cardiovascular disease, cardiovascular disease mortality, and all-cause mortality in a general population of middle-aged adults. J Am Heart Assoc. (2019) 8:e012865. doi: 10.1161/JAHA.119.012865, 31387433 PMC6759882

[47] Wright N Wilson L Smith M Duncan B McHugh P. The BROAD study: a randomised controlled trial using a whole food plant-based diet in the community for obesity, ischaemic heart disease or diabetes. Nutr Diabetes. (2017) 7:e256. doi: 10.1038/nutd.2017.3, 28319109 PMC5380896

[48] Ruiz-Canela M Zazpe I Shivappa N Hébert JR Sánchez-Tainta A Corella D . Dietary inflammatory index and anthropometric measures of obesity in a population sample at high cardiovascular risk from the PREDIMED (PREvención con DIeta MEDiterránea) trial. Br J Nutr. (2015) 113:984–95. doi: 10.1017/S0007114514004401, 25720588 PMC4870040

[49] Ji M Hong X Chen M Chen T Wang J Zhang N. Dietary inflammatory index and cardiovascular risk and mortality: a meta-analysis of cohort studies. Medicine (Baltimore). (2020) 99:e20303. doi: 10.1097/MD.0000000000020303, 32443378 PMC7253850

[50] Zahedi H Djalalinia S Asayesh H Mansourian M Esmaeili Abdar Z Mahdavi Gorabi A . A higher dietary inflammatory index score is associated with a higher risk of incidence and mortality of cancer: a comprehensive systematic review and meta-analysis. Int J Prev Med. (2020) 11:15. doi: 10.4103/ijpvm.IJPVM_332_18, 32175055 PMC7050224

[51] Abbasalizad Farhangi M Vajdi M Fathollahi P. Dietary total antioxidant capacity (TAC), general and central obesity indices and serum lipids among adults: an updated systematic review and meta-analysis. Int J Vitam Nutr Res. (2022) 92:406–22. doi: 10.1024/0300-9831/a000675, 32777987

[52] Pourreza S Khademi Z Mirzababaei A Yekaninejad MS Sadeghniiat-Haghighi K Naghshi S . Association of plant-based diet index with inflammatory markers and sleep quality in overweight and obese female adults: a cross-sectional study. Int J Clin Pract. (2021) 75:e14429. doi: 10.1111/ijcp.14429, 34081826

[53] Cheng E Hong CC Ergas IJ Caan BJ Kwan ML Roh JM . Plant-based diet, inflammation biomarkers and body composition among women with breast cancer: the pathways study. Br J Nutr. (2025) 133:1309–19. doi: 10.1017/S0007114525000856, 40384274 PMC12284747

[54] Aljuraiban GS Gibson R Al-Freeh L Al-Musharaf S Shivappa N Hébert JR . Associations among plant-based dietary indexes, the dietary inflammatory index, and inflammatory potential in female college students in Saudi Arabia: a cross-sectional study. J Acad Nutr Diet. (2022) 122:771–785.e8. doi: 10.1016/j.jand.2021.08.111, 34481119

[55] Baden MY Satija A Hu FB Huang T. Change in plant-based diet quality is associated with changes in plasma adiposity-associated biomarker concentrations in women. J Nutr. (2019) 149:676–86. doi: 10.1093/jn/nxy301, 30927000 PMC6461739

