# Supplementary material for: Associations of the unhealthy plant-based diet index with hsCRP and BMI in Korean adults
Source: Front Nutr. 2026 Aug 4;13:1836622. doi: 10.3389/fnut.2026.1836622 (PMC13481229; doi:10.3389/fnut.2026.1836622)
Supplement: Supplementary file 1 [file Supplementary_file_1.docx]

| **Table S1.** 19-food group classification for the unhealthy Plant-based Diet Index (uPDI) | | |
| --- | --- | --- |
| **Food group** | **Category** | **Representative foods** |
| 1. Whole grains | Healthy plant | Brown rice, barley, oats |
| 2. Fruits | Healthy plant | Apple, pear, tangerine, banana |
| 3. Vegetables | Healthy plant | Spinach, cabbage, radish, lettuce |
| 4. Nuts | Healthy plant | Peanuts, walnuts, chestnuts, almonds |
| 5. Legumes | Healthy plant | Soybeans, red beans, tofu, soy milk |
| 6. Vegetable oils | Healthy plant | Perilla oil, sesame oil, soybean oil |
| 7. Tea and coffee | Healthy plant | Green tea, black tea, coffee |
| 8. Fruit juices | Less healthy plant | Orange juice, apple juice |
| 9. Refined grains | Less healthy plant | White rice, noodles, white bread |
| 10. Potatoes | Less healthy plant | Potato |
| 11. Sugar-sweetened beverages | Less healthy plant | Carbonated soda, sports drinks |
| 12. Sweets and desserts | Less healthy plant | Candy, cookies, ice cream, cake |
| 13. Salty foods | Less healthy plant | Kimchi, salted vegetables, instant noodles |
| 14. Animal fats | Animal | Lard, butter |
| 15. Dairy products | Animal | Milk, yogurt, cheese |
| 16. Eggs | Animal | Chicken eggs |
| 17. Fish and seafood | Animal | Mackerel, anchovy, squid, shrimp |
| 18. Meats | Animal | Beef, pork, chicken |
| 19. Miscellaneous animal-based foods | Animal | Animal-based broths/soups, gelatin |
| ***** Food group classification was based on the 18-food group plant-based diet index framework of Satija et al. (2016), with a salty foods group additionally incorporated to reflect the characteristics of the Korean diet, yielding a total of 19 food groups, and KNHANES 24-hour dietary recall food and dish codes were mapped to each group. | | |
