# Supplementary material for: Associations of the unhealthy plant-based diet index with hsCRP and BMI in Korean adults
Source: Front Nutr. 2026 Aug 4;13:1836622. doi: 10.3389/fnut.2026.1836622 (PMC13481229; doi:10.3389/fnut.2026.1836622)
Supplement: Supplementary file 2 [file Supplementary_file_2.docx]

| **Table S2.** Baseline characteristics of included (n=5,610) and excluded participants due to missing hsCRP values (n=1,280), stratified by sex**^a^** | | | | | | |
| --- | --- | --- | --- | --- | --- | --- |
| **Variables** | **Men** | | | **Women** | | |
|  | **Included**  **(n=2,607)** | **Excluded**  **(n=429)** | **P-value^b^** | **Included**  **(n=3,003)** | **Excluded**  **(n=851)** | **P-value** |
| **Age (years)** | 42.8 ± 0.3 | 40.1 ± 0.7 | **<0.001** | 44.6 ± 0.3 | 41.3 ± 0.5 | **<0.001** |
| **Household income (n, %)^c^** |  |  | 0.124 |  |  | 0.145 |
| Low | 143 (5.1) | 37 (8.3) |  | 193 (5.6) | 63 (8.1) |  |
| Middle-low | 306 (11.0) | 47 (9.8) |  | 474 (14.8) | 116 (13.4) |  |
| Middle | 554 (21.1) | 87 (19.7) |  | 706 (23.0) | 205 (23.1) |  |
| Middle-high | 740 (29.1) | 123 (30.0) |  | 810 (27.8) | 207 (25.6) |  |
| High | 860 (33.7) | 133 (32.2) |  | 817 (28.8) | 260 (29.8) |  |
| **Education level (n, %)** |  |  | 0.087 |  |  | **0.022** |
| ≤ Elementary school | 67 (1.8) | 9 (1.4) |  | 165 (4.1) | 31 (3.0) |  |
| Middle school graduate | 121 (3.6) | 13 (2.9) |  | 201 (5.5) | 42 (4.1) |  |
| High school graduate | 932 (36.0) | 181 (42.5) |  | 1,175 (39.9) | 305 (36.1) |  |
| ≥ College or university | 1,462 (58.6) | 220 (53.2) |  | 1,440 (50.5) | 462 (56.8) |  |
| **Physical activity (n, %)** |  |  | 0.383 |  |  | **0.015** |
| Yes | 1,310 (55.3) | 216 (58.0) |  | 1,386 (49.8) | 425 (55.8) |  |
| No | 1,130 (44.7) | 175 (42.0) |  | 1,479 (50.2) | 364 (44.2) |  |
| **Smoking status (n, %)** |  |  | 0.069 |  |  | 0.813 |
| Yes | 898 (33.6) | 117 (28.5) |  | 153 (5.3) | 45 (5.6) |  |
| No | 1,696 (66.4) | 307 (71.5) |  | 2,840 (94.7) | 799 (94.4) |  |
| **Alcohol consumption (n, %)** |  |  | 0.205 |  |  | 0.064 |
| Yes | 1,826 (69.7) | 299 (73.1) |  | 1,423 (49.3) | 445 (53.2) |  |
| No | 770 (30.3) | 125 (26.9) |  | 1,570 (50.7) | 399 (46.8) |  |
| **Menopausal status^d^ (n, %)** |  |  |  |  |  | **<0.001** |
| Yes |  |  |  | 1,087 (35.0) | 228 (24.1) |  |
| No |  |  |  | 1,597 (65.0) | 530 (75.9) |  |
| **Energy intake (kcal)** | 2,164.0 ± 18.0 | 2,110.6 ± 40.5 | 0.240 | 1,609.0 ± 13.3 | 1,564.4 ± 23.0 | 0.101 |
| **BMI, kg/m^2^** | 25.7 ± 0.1 | 23.4 ± 0.2 | **<0.001** | 24.1 ± 0.1 | 21.8 ± 0.1 | **<0.001** |
| **^a^** Values are presented as mean ± standard error for continuous variables and n (%) for categorical variables, estimated using survey-weighted procedures (PROC SURVEYREG, PROC SURVEYFREQ, PROC SURVEYMEANS) to account for the complex, multistage probability sampling design of KNHANES.  **^b^** P-values were calculated using survey-weighted linear regression (PROC SURVEYREG) for continuous variables and the Rao-Scott chi-square test (PROC SURVEYFREQ) for categorical variables.  **^c^** Percentages for categorical variables are row percentages calculated among participants with non-missing responses for each variable; participants with missing or no-response values for a given variable were excluded from that variable's percentage calculation.  **^d^** Menopausal status was assessed only in women (included n = 2,684, excluded n = 758).  **^*^** Comparisons between included and excluded participants were performed separately for men and women.  **^*^** Bold p-values indicate statistical significance (p < 0.05). | | | | | | |
